# Instrumental variable analysis using offspring BMI in childhood as an indicator of parental BMI in relation to mortality

**DOI:** 10.1038/s41598-021-01352-w

**Published:** 2021-11-17

**Authors:** Kim Blond, David Carslake, Line Klingen Gjærde, Dorte Vistisen, Thorkild I. A. Sørensen, George Davey Smith, Jennifer L. Baker

**Affiliations:** 1 Center for Clinical Research and Prevention, Bispebjerg and Frederiksberg Hospital, The Capital Region, Copenhagen, Denmark; 2 Medical Research Council (MRC) Integrative Epidemiology Unit, University of Bristol, Bristol, UK; 3 Population Health Sciences, Bristol Medical School, University of Bristol, Bristol, UK; 4 Children’s Hospital Copenhagen and Juliane Marie Centre, Rigshospitalet, The Capital Region, Copenhagen, Denmark; 5 Steno Diabetes Center Copenhagen, Gentofte, Denmark; 6 Department of Public Health, Faculty of Health and Medical Sciences, Novo Nordisk Foundation Center for Basic Metabolic Research, University of Copenhagen, Copenhagen, Denmark

**Keywords:** Risk factors, Epidemiology, Obesity

## Abstract

Childhood BMI shows associations with adult mortality, but these may be influenced by effects of ill health in childhood on BMI and later mortality. To avoid this, we used offspring childhood BMI as an instrumental variable (IV) for own BMI in relation to mortality and compared it with conventional associations of own childhood BMI and own mortality. We included 36,097 parent–offspring pairs with measured heights and weights from the Copenhagen School Health Records Register and register-based information on death. Hazard ratios (HR) were estimated using adjusted Cox regression models. For all-cause mortality, per zBMI at age 7 the conventional HR = 1.07 (95%CI: 1.04–1.09) in women and 1.02 (95%CI: 0.92–1.14) in men, whereas the IV HR = 1.23 (95%CI: 1.15–1.32) in women and 1.05 (95%CI: 0.94–1.17) in men. Per zBMI at age 13, the conventional HR = 1.11 (95%CI: 1.08–1.15) in women and 1.03 (95%CI: 0.99–1.06) in men, whereas the IV HR = 1.30 (95%CI: 1.19–1.42) in women and 1.15 (95%CI: 1.04–1.29) in men. Only conventional models showed indications of J-shaped associations. Our IV analyses suggest that there is a causal relationship between BMI and mortality that is positive at both high and low BMI values.

## Introduction

Children, and especially adolescents, with obesity have a higher mortality rate than children with normal weight^1–5^. Further, there are indications that low body mass index (BMI) values in childhood are associated with increased adult mortality^6,7^ in a manner similar to associations between adulthood BMI and mortality^8^. These associations may be influenced by disease and general ill health that affect child BMI and adult mortality^9–26^. An alternate approach that can largely bypass this influence uses offspring BMI as instrumental variable (IV) for one’s parental BMI. This method effectively captures the association between the genetic variation in BMI and mortality. As such, the association between the IV of offspring BMI and parental mortality may, to a greater extent, represent the association in individuals whose BMI is not affected by ill health and may thus be better suited to examine the direction of the effects of different BMI levels on mortality.

Previous studies have used the IV of offspring BMI at adult ages to assess its associations with parental mortality. Collectively, these studies suggest that confounding by ill health may have a substantial influence on conventional associations between adult BMI and mortality^27–29^. The intergenerational association in BMI is caused by heritability of a mixture of exposures which differ by age as, for example, alcohol consumption and tobacco have minimal roles in child BMI, but constitute a part of the heritability of adult BMI^30,31^. As such offspring childhood BMI as the IV likely represents a somewhat different exposure than offspring adulthood BMI does. Furthermore, the use of offspring childhood BMI as an IV may on average represent a shift in BMI trajectory earlier in life than using offspring adulthood BMI as an IV does.

Therefore, with the aim of examining the effect of zBMI on mortality, we compared conventional estimates with IV estimates using offspring childhood zBMI as an IV for parental zBMI, and we extend the previous use of offspring BMI as an IV by examining the shape of BMI to mortality relations in IV analyses.

## Methods

The Copenhagen School Health Records Register (CSHRR)^32^ was used to obtain measured values of weight and height for parents and their offspring from mandatory health examinations conducted by doctors and nurses. The linkage of parents to offspring was done via a personal identification number, which all Danish citizens have been assigned since 1968^33,34^. For individuals born before the personal identification numbers were introduced, these numbers were assigned based on the persons forename(s) and surname, sex and date of birth. A personal identification number was available for approximately 89% of the children in CSHRR, with the main reasons for missingness being death or emigration before the introduction of the number. Parent–offspring pairs were identified in the national Fertility Database^35^. Based on the limits of family identifications in the Fertility Database, we restricted our eligible population to offspring born after 1952. In the CSHRR, 56,149 mother–offspring and 47,230 father-offspring pairs were identified. The analytic sample was limited to parent–offspring pairs in which the parent and child had BMI values available at both 7 and 13 years of age. We randomly chose only one offspring per parent for inclusion in the dataset. With these requirements, the analytic sample consisted of 36,097 parent–offspring pairs of whom 19,869 were mother–offspring pairs and 16,228 were father-offspring pairs.

All-cause mortality and cause-specific mortality were retrieved from 1970 onwards via linkages with the Danish Register of Vital Statistics and the Danish Cause of Death Register. International Classification of Disease (ICD) codes were used to define the causes of death. Until 1994 ICD-8 codes were used, and thereafter ICD-10 codes were used (ICD-9 was not used in Denmark). The primary outcomes were all-cause, CVD and cancer mortality, and the secondary outcomes were mortality from coronary heart disease (CHD), respiratory disease, gastrointestinal disease, urogenital disease, infectious disease, neurological disease, musculoskeletal disease, endocrine disease, mental illness and injuries (Supplementary Table 1).

Using an internal age-, sex-specific reference from a period (1955–1960) when the prevalence of overweight and obesity was low, BMIs were transformed into sex and age-specific z scores (hereafter, zBMI) using the Lambda, Median, Sigma (LMS) method^36,37^. Height measurements were also transformed to z scores using the LMS method based upon internal sex-, age- and birth cohort-specific references (5-year intervals). If the height and weight measurements were taken at exactly age 7 or exactly age 13, we used the z score value calculated from these measurements. Otherwise, z-scores for exact ages 7 and 13 were calculated by interpolating two measurements between ages 6–8 and 12–14, respectively, or by extrapolation if height and weight was only measured one time during either of these two age-spans.

We estimated conventional hazard ratios (HR) per zBMI with Cox proportional hazards regression models using own zBMI at ages 7 and 13 in separate analyses. Parental age was the time axis with entry at the child’s date of birth. Parental person-time was censored if they emigrated, were lost to follow-up or died. We adjusted for parental sex, parental birth cohort (1930–39, 1940–49, or 1950–83) and offspring birth cohort (1952–62, 1963–73, or 1974–1996) and parental and offspring zheight. Via stratification, we allowed the baseline hazard to vary for each level of the covariate for the categorical variables (parental sex and parental and offspring birth cohort), except when examining interactions between BMI and these variables. We adjusted for zheight because it is associated with both zBMI (Supplementary Table 2) and adult mortality. Non-linear conventional associations (adjusted for the covariates previously mentioned) were also modelled with restricted cubic splines with knots at the 5th, 27.5th, 50th, 72.5th and 95th percentiles. Non-linearity was tested with a Wald-type test of the coefficients for the spline terms (except the coefficient for spline term corresponding to the linear term) being equal to zero^38^. Linear spline analyses were conducted with a knot point at zBMI = 0.

Offspring zBMIs at ages 7 and 13 were used as the IVs. For unadjusted IV estimates to be causal effects, four assumptions need to be made: (1) offspring zBMI and parental zBMI must be associated, (2) no direct effect of offspring zBMI on parental mortality, (3) no common causes of offspring zBMI and parental mortality, and (4) monotonicity: if a given genotype increases childhood BMI in one individual it must not decrease BMI in another individual. We assume that the association between parental zBMI and offspring zBMI is due to their shared genetics (Supplementary Fig. 1), and we quantify these associations via linear regression models. Assumption 3 is of primary concern as it is likely not fulfilled, and to partially account for this, we include adjustments for the covariates that were also included in the conventional Cox proportional hazards models. Although offspring BMI may not be a completely valid IV, we argue that using offspring BMI as an IV is likely less biased than conventional analyses. As such the IV results are of primary interest from a causal viewpoint, and the conventional associations will be evaluated by their ability to represent the IV findings.

IV HRs were derived by exponentiating the ratio of 1) the natural logarithm of the HR of all-cause and cause specific deaths per z-score of child BMI using Cox proportional hazards regression (numerator) and 2) the mean difference in parental zBMI per z-score of offspring BMI from a linear regression (denominator). The numerator was as the conventional models except offspring zBMI was included instead of parental zBMI. The denominator was adjusted for the same variables which were in the numerator. Although we scale (via the denominator) our IV estimates to represent (parental) zBMI at ages 7 and 13 as the exposures, caution is needed in the interpretation^39^ as neither our conventional or our IV estimates apply causally to specific ages since it is likely that genetic variants that affect BMI in childhood and also directly affect adult BMI. Due to the scaling via the denominator, IV HRs are expressed per parental zBMI, which is similar to conventional analyses—therefore all HRs (except Supplementary Table 7) are simply denoted as per zBMI. Taylor series expansions were used to calculate confidence intervals (CI)^40^. In analyses combining the parents, clustering by offspring identity was taken into account by using robust standard errors. The difference between the HRs from log-linear IV and conventional models was evaluated with a Durbin-Wu-Hausman test^41^. Interactions with birth cohort and parental sex were tested with a Z-test of the included product terms in the numerator.

To generate non-linear IV plots, non-linear IV associations were modelled by estimating local IV estimates in strata between approximately the 1.5, 10, 27.5, 50, 72.5, 90 and 98.5th percentiles of the instrument-free exposure, which is the residual from when parental zBMI is regressed on offspring zBMI^42^. Corresponding quantiles of the original exposure were used in piecewise linear plots made by joining the local IV estimates^43^. The local IV estimates were estimated under the assumption that the association between the instrument and exposure was constant across exposure levels. We tested non-linearity in the IV association and in the denominator by conducting meta-regressions with stratum-specific IV estimates or stratum-specific parent–offspring zBMI associations (i.e. the IV denominator) as the dependent variable and the stratum-specific mean parental zBMI as the independent variable. CIs were generated by bootstrapping with 1,000 repetitions. We conducted analyses to examine the consistency of the results without height adjustment and across parental and offspring sex, birth cohort and age during follow-up (the proportional hazards assumption), see Supplementary methods for details.

According to Danish law, register-based research projects such as our study that use pre-existing information and do not contact individuals do not require ethical approval or written consent. The study was approved and registered with the Danish Data Protection Agency under the permission given to the Capital Region of Denmark (Approval number 2007-58-0015).

### Ethics approval

All procedures performed in studies involving human participants were in accordance with the ethical standards of the institution and national research committee and with the 1964 Helsinki declaration and its later amendments or comparable ethical standards. The study was approved by the Danish Data Protection Agency.

### Consent to participate and for publication

According to Danish law, informed consent is not required for purely register-based research of pre-existing data. Thus, for this type of study formal consent is not required.

## Results

In our study sample, the offspring were born from 1953 to 1996, whereas the mothers were born from 1930 to 1981 and the fathers from 1930 to 1979. The mothers are on average 25 years old (IQR = 21–28) and the fathers were on average 27 years old (IQR = 23–30) when they had the child included in our study. During follow-up, 11,641 parents died. The median follow-up time was 40 years. Median ages at entry and exit were 29 years and 69 years, respectively. Cardiovascular disease and cancer were the most common causes of death (Supplementary Table 3). Table 1 shows the levels of potential confounders by levels offspring BMI at age 7 (by age 13 in Supplementary Table 4), and it shows that birth year and zheight were higher for higher levels of offspring zBMI categories. In parents, the median BMI was 15.3 at age 7 and 18.0 at age 13, and in offspring the median BMI was 15.5 at age 7 and 18.3 at age 13 (additional details on the BMI distribution are in Supplementary Table 5).

**Table 1:** Years of birth and parental age at birth of offspring by categories of offspring zBMI at age 7 years.

Group	Characteristic	Categories of offspring zBMI at age 7							Overall
		< − 2	− 2 to > − 1	− 1 to < − 0.5	− 0.5 to < 0.5	0.5 to < 1	1 to < 2	≥ 2	All offspring
Offspring (n = 17,715 girls, n = 18,382 boys)	Birth year	1970^2^	1969	1969	1970	1972	1973	1978	1968 (1962–1985)^1^
	Zheight at age 7	− 0.46^2^	− 0.29	− 0.20	− 0.10	0.08	0.29	0.71	− 0.03
Mothers (n = 19,869)	Birth year	1945^2^	1944	1944	1945	1946	1948	1951	1944 (1937–1952)^1^
	Age at birth (y)	25^2^	25	25	25	25	25	26	24 (21–28)^1^
	Zheight at age 7	− 0.35^2^	− 0.29	− 0.22	− 0.18	− 0.11	− 0.02	0.08	− 0.16
Fathers (n = 16,228)	Birth year	1944^2^	1943	1944	1944	1946	1947	1951	1943 (1937–1952)^1^
	Age at birth (y)	27^2^	27	27	27	27	28	28	26 (23–30)^1^
	Zheight at age 7	− 0.34^2^	− 0.24	− 0.18	− 0.15	− 0.11	− 0.04	0.05	− 0.14

^1^ Median (IQR).

^2^ Mean.

The denominators and (exponentiated) numerators used to calculate the IV estimates are shown in Supplementary tables 6 and 7. Each unit higher offspring zBMI at age 7 was associated with 0.28 (95% CI: 0.27–0.29) units higher parental zBMI at age 7 (partial F-statistic = 3902 with 36,083 degrees of freedom and partial R squared = 0.10) in a model that was adjusted for all covariates including zheight. For the association between offspring zBMIs at age 13 and parental zBMI at age 13, the coefficient was 0.30 (95% CI: 0.29–0.30, partial F-statistic = 4595 with 36,083 degrees of freedom and partial R squared = 0.10) (Supplementary Table 6). The associations were stronger in mother–offspring pairs than in father-offspring pairs. When associations between offspring zBMI at 7 and parental zBMI at 13, and vice versa, were examined, these coefficients were slightly lower. These associations showed little deviation from linearity throughout most of the BMI spectrum (Supplementary Figs. 2–7). The meta-regression also showed that the potential deviation from linearity was small as the coefficient for variation in the exposure-instrument slope as a function of mean zBMI in the strata of the instrument-free exposure in men was -0.004 (*p* for linear trend = 0.078) for zBMI at age 7 and 0.002 (*p* for linear trend = 0.427) at age 13. In women, the coefficient was 0.002 (p for linear trend = 0.281) at age 7 and 0.000 (*p* for linear trend = 0.937) at age 13.

**Figure 1:**
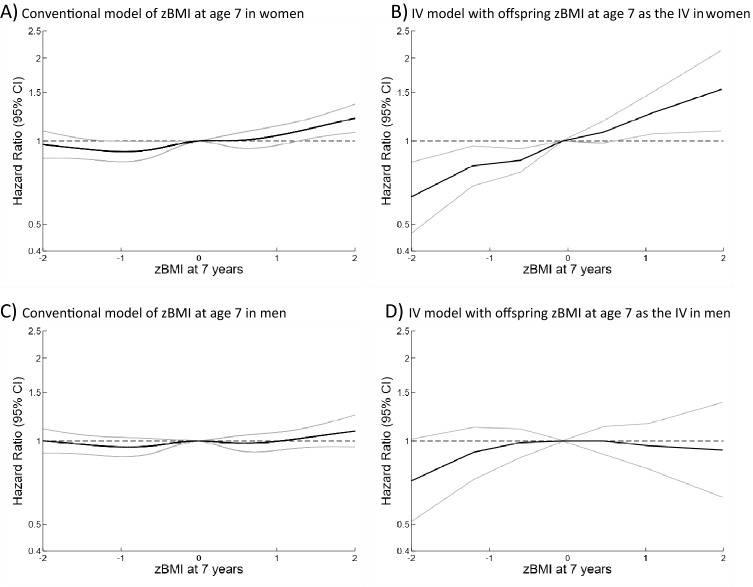
Association between zBMI at age 7 and all-cause mortality in men estimated by a conventional model and instrumental variable (IV) model. All models are adjusted for parental and offspring birth cohort, parental and offspring zheight.

**Figure 2:**
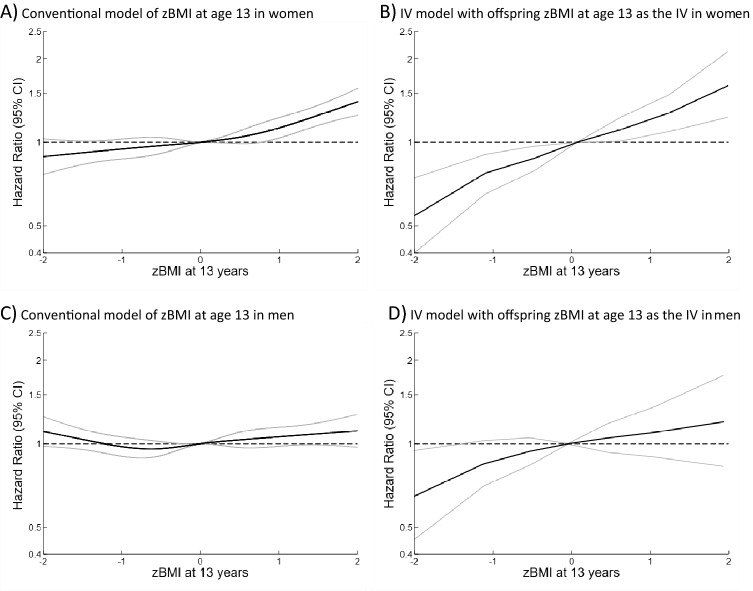
Association between zBMI at age 13 and all-cause mortality estimated by a conventional model and instrumental variable (IV) model. All models are adjusted for parental and offspring birth cohort, parental and offspring zheight.

In conventional Cox models, the all-cause mortality HR per zBMI was between 1.03 (95% CI: 1.01–1.05) and 1.07 (95%CI: 1.04–1.09) dependent upon zheight adjustment and age at the BMI measurement (Tables 2 and 3 and Supplementary tables 8 and 9). The hazard ratios were slightly higher for zBMI at age 13 than at age 7. All-cause mortality HRs per zBMI were higher in women than men (*p*-values for interaction between sex and zBMI: *p* = 0.068 for zBMI at age 7 and *p* < 0.001 for zBMI at age 13), whereas the evidence was weaker for CVD (p-values for interaction between sex and zBMI: *p* = 0.124 for zBMI at age 7 and *p* = 0.629 for zBMI at age 13), and cancer mortality (p-values for interaction between sex and zBMI: *p* = 0.293 for zBMI at age 7 and *p* = 0.079 for zBMI at age 13). HRs per zBMI were positive for mortality from CHD, respiratory disease, urogenital disease, infectious disease, nervous system disease, musculoskeletal disease, endocrine disease and external causes (Supplementary Tables 10 and 11). Differences by parental birth cohorts were most apparent for CVD mortality, and there was limited evidence for non-proportional hazards (Supplementary Tables 12 and 13). In analyses allowing for non-linearity, there was evidence of positive associations with all-cause mortality only at zBMI > 0 (Figs. 1 and 2 and Supplementary Table 14), and the shape of the associations differed by parental sex (Figs. 1–2 and Supplementary Figs. 8–15).

**Table 2:** Associations between zBMI at age 7 and adult mortality: hazard ratios (95% confidence intervals) per zBMI estimated from conventional analyses of own zBMI and from analyses using offspring zBMI at age 7 as instrumental variable (IV).

Cause of death	Models^1^		*P*-values		
	Conventional (C)	Offspring zBMI at age 7 as IV (IV)	*P*_IV vs C_^2^	*P*_non-linearity for own zBMI_^3^	*P*_non-linearity in IV_^4^
**Women and men**					
All-cause	1.04 (1.02–1.06)	1.13 (1.05–1.21)	0.013	0.020	0.350
Cardiovascular disease	1.06 (1.03–1.10)	1.21 (1.08–1.35)	0.022	0.062	0.343
Cancer	1.04 (1.00–1.07)	1.06 (0.95–1.18)	0.733	0.319	0.092
**Women**					
All-cause	1.07 (1.04–1.09)	1.23 (1.15–1.32)	< 0.001	0.105	0.880
Cardiovascular disease	1.09 (1.04–1.13)	1.36 (1.20–1.53)	< 0.001	0.188	0.081
Cancer	1.08 (1.04–1.12)	1.11 (1.00–1.23)	0.595	0.711	0.481
**Men**					
All-cause	1.02 (0.99–1.05)	1.05 (0.94–1.17)	0.594	0.408	0.321
Cardiovascular disease	1.04 (0.99–1.08)	1.11 (0.94–1.32)	0.402	0.238	0.776
Cancer	1.02 (0.97–1.07)	1.01 (0.84–1.21)	0.941	0.543	0.215

^1^Adjusted for offspring and parental birth cohort, parental and offspring zheight and parental sex.

^2^*P*-values from Durbin-Wu-Hausman test.

^3^*P*-values from a Wald test of non-linearity in the conventional association.

^4^Linear trend test from a meta-regression of stratum specific IV estimates on the stratum-specific mean zBMI.

*BMI* body mass index, *IV* instrumental variable.

**Table 3:** Associations between zBMI at age 13 and adult mortality: hazard ratios (95% confidence intervals) per zBMI estimated from conventional analyses of own zBMI and from analyses using offspring zBMI at age 13 as instrumental variable (IV).

Cause of death	Model^1^		*P*-values		
	Conventional (C)	Offspring zBMI at age 13 as IV (IV)	*P*_IV vs C_^2^	*P*_non-linearity for own zBMI_^3^	*P*_non-linearity in IV_^4^
**Women and men**					
All-cause	1.07 (1.04–1.09)	1.22 (1.14–1.31)	< 0.001	0.003	0.670
Cardiovascular disease	1.16 (1.11–1.20)	1.36 (1.22–1.53)	0.002	0.045	0.681
Cancer	1.03 (0.99–1.07)	1.08 (0.97–1.20)	0.346	0.989	0.796
**Women**					
All-cause	1.11 (1.08–1.15)	1.30 (1.19–1.42)	< 0.001	0.085	0.785
Cardiovascular disease	1.18 (1.11–1.24)	1.49 (1.28–1.74)	0.001	0.114	0.613
Cancer	1.06 (1.01–1.11)	1.13 (0.99–1.29)	0.304	0.724	0.723
**Men**					
All-cause	1.03 (0.99–1.06)	1.15 (1.04–1.29)	0.027	0.047	0.516
Cardiovascular disease	1.15 (1.09–1.20)	1.30 (1.10–1.54)	0.122	0.133	0.664
Cancer	0.99 (0.94–1.05)	1.02 (0.85–1.23)	0.747	0.874	0.397

^1^Adjusted for offspring and parental birth cohort, parental and offspring zheight and parental sex.

^2^*P*-values from Durbin-Wu-Hausman test.

^3^*P*-values from a Wald test of non-linearity in the conventional association.

^4^Linear trend test from a meta-regression of stratum specific IV estimates on the stratum-specific mean zBMI.

*BMI* body mass index, *IV* instrumental variable.

In IV analyses of parents combined, the all-cause mortality HR per zBMI ranged from 1.07 (95% CI: 1.00–1.14) to 1.25 (95% CI: 1.16–1.36) dependent upon zheight adjustment and age at the BMI measurement (Tables 2 and 3 and Supplementary Tables 8, 9, 15 and 16). The associations were stronger when adjusting for zheight. The IV estimates were stronger than the conventional estimates for all-cause, CVD and several other causes of mortality, with the only exception being cancer mortality where neither the IV nor the conventional model showed clear associations (Tables 2 and 3 and Supplementary Tables 10, 11 and 14). The HRs per zBMI were generally higher in women than in men, but the evidence for sex interactions was not strong (p-values for interaction between sex and zBMI at ages 7 and 13, respectively: *p* = 0.102 and 0.147 for all-cause mortality, *p* = 0.227 and 0.348 for CVD mortality and *p* = 0.506 and 0.377 for cancer mortality). The associations were generally stronger when using BMI at age 13 as the IV than when using BMI at age 7 as the IV (Supplementary Tables 15 and 16). When using BMI at age 7 as the IV, the point estimates were higher when using girls’ BMI than when using boys’ BMI as the IV (Supplementary Table 17), particularly for cancer mortality. When using BMI at age 13 as the instrument, the estimates did not appear to differ whether using boys’ or girls BMI as the IV (Supplementary Table 18). There was little evidence of deviations from log-linearity in IV associations (Tables 2 and 3, Figs. 1 and 2, Supplementary Figs. 8–15 and Supplementary Table 14). In analyses of all-cause mortality allowing for non-linearity, the IV slopes at zBMI < 0 contrasted with the conventional slopes, whereas there was no consistent evidence of a difference between the IV and the conventional slopes at zBMI > 0 (Figs. 1 and 2, Supplementary Table 14).

## Discussion

Among 36,097 parent–offspring pairs, we found positive associations (per zBMI) at age 7 and 13 for all-cause mortality, CVD mortality and cancer mortality. For all-cause mortality, the associations were stronger in women than men. For all-cause mortality and CVD mortality, we found stronger HRs per zBMI in IV models than in conventional models. Although we found evidence for non-linearity in conventional models, we found less evidence of deviations from linearity in IV models. Specifically, indications of inverse associations at low BMI levels in conventional models were virtually absent in IV models. As such, the IV and the conventional analyses broadly agree that linear mortality estimates are positive, but they do not agree on the magnitude of the linear estimates for all-cause and CVD mortality which may partly be due to differential shapes of the associations between the two models. Our findings imply that shifting the BMI distribution towards lower BMI levels may be more beneficial for mortality than suggested by conventional analyses, and that it may be beneficial across a larger spectrum of BMI values than is suggested by conventional analyses.

Several previous conventional analyses of childhood BMI and mortality in adulthood only compare overweight or obesity with normal weight^1,2,4,5^, but some studies examined BMI in a more detailed way^3,6,7,44^. In these studies, a J-shape or a U-shape was found, but the sub-groups for which it was present differed among the studies. In analyses of BMI in 15-year-old British children, a U-shape was found in girls and an inverse association was found in boys^6^, whereas a Norwegian study of BMI at ages 14–19 found a J-shape in boys but not in girls^7,44^. Another British study found a J-shape among children aged 2–8 years at the BMI measurement, but not among children among children aged 8–14 years^3^. In contrast, J-shapes in our conventional analyses were present at age 7 in boys and girls and at age 13 only in boys. One of these previous studies examining the shape of the mortality association across the BMI spectrum adjusted for SEP and found no impact of this adjustment, but none of these studies accounted for ill health affecting childhood BMI suggesting this may be an explanation of the discrepancy between the conventional findings and the IV findings.

Our finding of a positive association at the lower BMI levels with mortality in the IV analyses indicates that the flat or inverse association between childhood BMI and adult mortality at the lower BMI levels in some of the conventional analyses may be due to underlying ill health. The difference in the direction of the estimates at the lower BMI levels is of particular interest. Although the magnitude of the estimates could differ if adult zBMI was analyzed as the exposure, the direction of the IV estimate is not influenced by the denominator (the association between offspring BMI and parental BMI) as it most likely is always above zero. One interpretation of the triangulation^45^ of the overall pattern of results from these analyses with previously reported data from studies of offspring BMI and parental mortality is that the naïvely estimated association between BMI and mortality is distorted by factors that increase mortality risk—including disease processes—lowering BMI^27–29^. The difference between all-cause mortality estimates between IV and conventional models appeared to be largely driven by CVD mortality whereas cancer mortality was much more similar between IV and conventional model^9–16^. However, although influence by ill health is more plausible in conventional models than in IV models, we cannot preclude that parental disease, via its potential effects on the parent’s general life circumstances, also affects offspring BMI.

Our IV models likely avoid influence by ill health, however they do not avoid confounding by parental covariates which could affect offspring BMI. A concern in this regard is that any bias in the association between offspring BMI and parental mortality will be inflated in inverse proportion to the association between own BMI and offspring BMI in the IV models^46^. Further, if there is a causal effect of parental BMI on offspring BMI, this would introduce confounding by the same confounders that also bias the conventional associations. A potential effect of parental BMI in childhood on offspring BMI in childhood would presumably, to some degree, be mediated by parental adult BMI. Although some^47–49^, but not all^50^, studies of bariatric surgery in the mother have found that it is associated with a reduced risk of obesity in the offspring, MR studies do not find an effect of maternal BMI during pregnancy on offspring adiposity at ages 7–18^51,52^. One potential confounder in the IV analyses is smoking as there is evidence that maternal smoking can affect birth weight, maternal smoking is associated with offspring BMI in childhood^53^ and as second hand smoking may affect BMI in children^54^. However, a genetically predicted higher BMI appears to cause a higher level of smoking, suggesting that the association between parental smoking and offspring BMI may not necessarily imply confounding^55^. Another factor worth considering is parental socioeconomic position (SEP). In Danish children born more recently than those in our study, parental SEP is inversely associated with child BMI^56–58^. Further, in Danish adoptees born 1924–1947, their adult BMI was inversely associated with the adoptive father’s SEP^59^. However, results from twin and adoption studies on the heritability of BMI and eating behaviors suggest that there is a decrease in the effect of the shared environment on BMI as children become adolescents^60,61^. As such, parental covariates may have a larger influence on BMI at 7 than at 13 years, and parental covariates may thus have a larger influence when using offspring zBMI at 7 years as the IV than when using zBMI at 13 years as the IV. This may explain the differences in the results when using offspring zBMI at age 7 as the IV versus offspring zBMI at age 13 as the IV. It is more difficult to speculate about the effect of using male offspring zBMI versus female offspring zBMI as the IV, as the results based on this stratification had wide confidence intervals and as we are not aware of literature on differential confounding structure in relation to these two IVs. In addition to differential influence by SEP between using BMI at age 7 and at age 13 as the instrument, tracking to parental adult BMI may also be differential as BMI at age 13 is more strongly associated with own adult BMI than BMI at age 7, and adult BMI is likely an important mediator of the effect of child BMI on adult mortality^62,63^.

Our conventional and our IV models share some limitations. Using BMI as a proxy for adiposity is a limitation since muscle mass and fat mass have largely opposing associations with mortality^64,65^ and as such the effect of fat mass is likely stronger than estimates for weight-based measures as recently found for the risk of type 2 diabetes^66^. The limitations of BMI and the differences in body composition between boys and girls^67^ may also be part of an explanation for the sex differences in our results. Although the associations between parental and offspring BMI appeared to be stable across time in the CSHRR^68,69^, temporal changes in body mass composition and thus its representation by BMI may also have affected our results. Additionally, the relation between BMI and mortality varies by ethnicity^70,71^, and due to the predominantly Caucasian ethnicity of our study population our estimates may not be generalizable to other ethnicities. Our estimates may also be affected by the uncertainty in the recording of causes of death^72^. An additional limitation is the lack of information on childhood ill health. Furthermore, although the CSHRR includes a large proportion of children in Copenhagen, selection bias may have affected our estimates since survival until having children and until the establishment of Danish Cause of Death Register in 1970 was required. A limitation of our comparisons between the IV and conventional estimates is that under the monotonicity assumption the effect potentially identified by IV estimates may be different from the average causal effect in the entire study population^73^.

The strengths of this study include that the heights and weights used to calculate BMI were measured in a standardized manner by doctors and nurses. Our ascertainment of the outcomes was register-based. We used a one-sample design, which may be more robust to survivor bias than a two-sample design^74^. Our data was well suited for using offspring BMI as an IV for own BMI as we used a validated register for family linkage. We identified 36,097 parent–offspring pairs, however we cannot preclude potential non-paternity which may partially explain our observation of a stronger association between parental zBMI and offspring zBMI in mothers than in fathers. We were able to provide precise estimates as 11,641 deaths occurred in our population, and we examined non-linearity in the associations.

Our IV analyses suggest that there is a causal relationship between BMI and mortality that is positive at both high and low BMI values.

## Supplementary Information

Supplementary Information.

## Data Availability

We will make the data (in de-identified form and to the best of our abilities given legal regulations) used in the manuscript available upon request.

## Code availability

Code is available to editors upon request either before or after publication for checking.
