# Supplementary material for: Instrumental variable analysis using offspring BMI in childhood as an indicator of parental BMI in relation to mortality
Source: Sci Rep. 2021 Nov 17;11:22408. doi: 10.1038/s41598-021-01352-w (PMC8599489; doi:10.1038/s41598-021-01352-w)
Supplement: Supplementary file 1 — Supplementary Information. [file 41598_2021_1352_MOESM1_ESM.docx]

**Supplementary table 1.** International classification of disease (ICD) codes used to define mortality

| Cause of death | ICD Version 8* | ICD Version10 |
| --- | --- | --- |
| Infectious | 001-136.9 | A15-A19.9  A20-A99.9  B00-B97.9  B99-B99.9 |
| Neoplasms | 140-199.9  200-239.9 | C00-D48.9 |
| Cardiovascular disease | 390-398.9  400-404.9  410-414.9  420-429.9  430-438.9  440-448.9  450-458.9 | I00-I99.9 |
| Coronary heart disease | 410-414.9  420-429.9 | I00-I25.9  I27-I27.9  I30-I52.9 |
| Respiratory disease | 460-474.9  480 -486.9  490 -493.9  500 -519.9 | J00 -J99.9 |
| Digestive disease | 520 -577.9 | K00 -K93.9 |
| Endocrine disease | 240 -246.9  250 -279.9 | E00 -E90.9 |
| Mental disease | 290 -315.9 | F00 -F99.9 |
| Urogenital disease | 580 -629.9 | N00 -N99.9 |
| Muscle disease | 710 -738.9 | M00 -M99.9 |
| Nervous system disease | 320 -389.9 | G00 -G99.9  H00 -H95.9 |
| External causes | 800 -949.9 | V01 -X59.9 |

*ICD-8 was used until 1993 and ICD-10 thereafter. ICD-9 was never used in Denmark.

**Methods**

Conventional models: Hazard ratios (HR) per zBMI not adjusted for zheight were estimated with Cox proportional hazards models adjusted for parental and offspring birth cohort and parental sex. Birth cohort and parental sex were included as stratification variables except when examining interaction between parental zBMI and the covariates. HRs per zBMI in women and men, separately, were estimated with Cox proportional hazards models adjusted for parental and offspring birth cohort, parental and offspring zheight and parental sex. HRs per zBMI for three parental birth cohorts (1930-39, 1940-49, and 1950-83) were estimated by Cox proportional hazards models adjusted for parental and offspring zheight, offspring birth cohort and parental sex. HRs per zBMI for three offspring birth cohorts (1952-62, 1963-73, and 1974-1996) were estimated by Cox proportional hazards models adjusted for parental and offspring zheight, parental birth cohort and parental sex. HRs per zBMI for three age groups (below age 60, from age 60 to 70, and above age 70) were estimated by Cox proportional hazards models adjusted for parental and offspring zheight, parental and offspring birth cohort and parental sex. Interactions with birth cohort and parental sex were tested with a Z-test of the included product terms in the Cox proportional hazards model. Proportional hazards were tested on the basis of Schoenfeld residuals from the Cox proportional hazards model.

The IV HRs were derived by exponentiating the ratio of 1) the natural logarithm of the HR of all-cause and cause specific deaths per z-score of offspring BMI using Cox proportional hazards regression (numerator) and 2) the mean difference in parental zBMI per z-score of offspring BMI from a linear regression (denominator). The numerator was as the conventional models except including offspring zBMI instead of parental zBMI. The denominator was adjusted for the same variables which were in the numerator. Birth cohort and parental sex were included as stratification variables except when examining interaction between parental zBMI and the covariates. Interactions with birth cohort and sex were tested with a Z-test of the included product terms in the numerator. Proportional hazards were tested on the basis of Schoenfeld residuals from the numerator. HRs per zBMI using zBMI in girls and zBMI in boys, respectively, as the IV were estimated with Cox proportional hazards models adjusted for parental and offspring birth cohort, parental and offspring zheight and parental and offspring sex.

**Supplementary Table 2.** Correlation coefficients between zBMI and zheight

|  | Correlation coefficient between zBMI and zheight (95% CI) | |
| --- | --- | --- |
| Group | Age 7 | Age 13 |
| Sons | 0.24 (0.22-0.25) | 0.32 (0.30-0.33) |
| Daughters | 0.25 (0.24-0.26) | 0.27 (0.26-0.29) |
| Fathers | 0.14 (0.13-0.16) | 0.31 (0.29-0.32) |
| Mothers | 0.17 (0.16-0.19) | 0.27 (0.26-0.28) |

**Supplementary table 3.** Causes of death among mothers and fathers*

| Cause of death | Parent | |
| --- | --- | --- |
|  | Mothers (n=19,869) | Fathers (n=16,228) |
| All-cause | 5,480 | 6,161 |
| Cardiovascular disease | 1,771 | 2,519 |
| Coronary heart disease | 1,189 | 1,868 |
| Cancer | 2,429 | 2,172 |
| Respiratory disease | 1,582 | 1,432 |
| Digestive disease | 584 | 842 |
| Urogenital disease | 239 | 260 |
| Infectious disease | 362 | 359 |
| Nervous system disease | 278 | 267 |
| Muscle disease | 105 | 63 |
| Endocrine disease | 542 | 741 |
| Mental disease | 703 | 1,096 |
| External causes | 148 | 247 |

*Causes of death are not mutually exclusive categories.

**Supplemental figure 1.** Directed acyclic graph showing the rationale (avoiding confounding by certain childhood diseases) for using offspring BMI as an imperfect (due to the possible confounders of the offspring BMI and parental mortality association) instrument for parental BMI in relation to parental mortality as the outcome

Offspring BMI

Childhood diseases and other possible confounders

(e.g. social class)

Parental mortality

Genetics

Parental BMI

Possible confounders (e.g. social class and smoking)

**Supplementary table 4.** Years of birth and parental age at birth of offspring by categories of offspring zBMI at age 13 years

|  | | | Categories of offspring zBMI at age 13 | | | | | | |
| --- | --- | --- | --- | --- | --- | --- | --- | --- | --- |
| Group | Characteristic | All offspring | <-2 | -2 to >-1 | -1 to <-0.5 | -0.5 to <0.5 | 0.5 to <1 | 1 to <2 | ≥2 |
| Offspring  (n=17,715 girls, n=18,382 boys) | Birth year | 1968 (1953-1996)^1^ | 1969 | 1968 | 1969 | 1970 | 1972 | 1974 | 1979 |
|  | Zheight at age 13 | 0.01^2^ | -0.70 | -0.47 | -0.29 | -0.01 | 0.25 | 0.36 | 0.45 |
| Mothers (n=19,869) | Birth year | 1944 (1937-1952)^1^ | 1944^2^ | 1943 | 1944 | 1945 | 1946 | 1948 | 1952 |
|  | Age at birth (y) | 24 (21-28)^1^ | 25^2^ | 24 | 24 | 24 | 25 | 25 | 26 |
|  | Zheight at age 13 | -0.11^2^ | -0.33^2^ | -0.30 | -0.24 | -0.12 | -0.02 | 0.01 | 0.09 |
| Fathers (n=16,228) | Birth year | 1943 (1937-1952)^1^ | 1943^2^ | 1943 | 1943 | 1944 | 1946 | 1948 | 1951 |
|  | Age at birth (y) | 26 (23-30)^1^ | 27^2^ | 27 | 27 | 27 | 27 | 28 | 29 |
|  | Zheight at age 13 | -0.08^2^ | -0.29^2^ | -0.24 | -0.18 | -0.10 | 0.02 | 0.04 | 0.05 |

^1^ Median (IQR)

^2^ Mean

**Supplementary table 5.** Sex-specific BMI values (kg/m^2^) at age 7 years by z-scores of our internal reference

|  | Z-scores and corresponding percentiles | | | | | | | | | | | | | |
| --- | --- | --- | --- | --- | --- | --- | --- | --- | --- | --- | --- | --- | --- | --- |
|  | -4  0.003 | -3  0.135 | -2  2.28 | -1.28  10 | -1  15.9 | 0  50 | 0.68  75 | 1  84.1 | 1.04  85 | 1.28  90 | 1.65  95 | 2  97.7 | 3  99.87 | 4  99.997 |
| Age (y) | Boys | | | | | | | | | | | | | |
| 7 | 12.0 | 12.6 | 13.4 | 14.0 | 14.3 | 15.4 | 16.3 | 16.8 | 16.8 | 17.2 | 17.8 | 18.5 | 20.8 | 24.1 |
| 13 | 12.7 | 13.6 | 14.7 | 15.6 | 16.0 | 17.8 | 19.4 | 20.3 | 20.4 | 21.1 | 22.5 | 24.0 | 30.7 | 48.1 |
|  | Girls | | | | | | | | | | | | | |
| 7 | 11.5 | 12.2 | 13.1 | 13.8 | 14.1 | 15.3 | 16.3 | 16.8 | 16.9 | 17.3 | 18.0 | 18.8 | 21.5 | 25.3 |
| 13 | 12.2 | 13.3 | 14.6 | 15.7 | 16.1 | 18.2 | 19.9 | 20.9 | 21.0 | 21.9 | 23.3 | 24.8 | 31.0 | 42.2 |

**Supplementary table 6.** Associations between offspring zBMI and parental zBMI at ages 7 and 13 years

|  | Parental zBMI | |
| --- | --- | --- |
| Offspring zBMI | 7 years | 13 years |
| 7 years | 0.28 (0.27-0.29)^1^ | 0.26 (0.26-0.27)^1^ |
| 13 years | 0.25 (0.25-0.26)^1^ | 0.30 (0.29-0.30)^1^ |
|  | Mother zBMI | |
|  | 7 years | 13 years |
| 7 years | 0.31 (0.30-0.32)^1^ | 0.29 (0.28-0.30)^1^ |
| 13 years | 0.29 (0.28-0.30)^1^ | 0.33 (0.32-0.35)^1^ |
|  | Father zBMI | |
|  | 7 years | 13 years |
| 7 years | 0.24 (0.22-0.25)^1^ | 0.22 (0.21-0.24)^1^ |
| 13 years | 0.21 (0.20-0.23)^1^ | 0.25 (0.24-0.26)^1^ |

^1^ Mean difference (95% CI) in parental zBMI per offspring zBMI, adjusted for offspring and parental birth cohort, parental and offspring zheight and parental sex (in the model combining mothers and fathers)

**Supplemental figure 2.** Parental zBMI at 7 years by 50 quantiles of offspring BMI at 7 years

**Supplemental figure 3**. Parental zBMI at 13 years by 50 quantiles of offspring BMI at 13 years

**Supplemental figure 4**. Maternal zBMI at 7 years by 50 quantiles of offspring BMI at 7 years

**Supplemental figure 5**. Paternal zBMI at 7 years by 50 quantiles of offspring BMI at 7 years

**Supplemental figure 6**. Maternal zBMI at 13 years by 50 quantiles of offspring BMI at 13 years

**Supplemental figure 7**. Paternal zBMI at 13 years by 50 quantiles of offspring BMI at 13 years

**Supplementary table 7.** Hazard ratios (95 % confidence intervals) from associations between offspring zBMI at age 7 and 13 and parental mortality (exponentiation of the numerator used to obtain the IV estimates)

|  | Models^1^ | |
| --- | --- | --- |
| Cause of death | zBMI at age 7 | zBMI at age 13 |
|  | Women and men | |
| All-cause | 1.03 (1.02-1.05) | 1.06 (1.04-1.08) |
| Cardiovascular disease | 1.05 (1.02-1.09) | 1.10 (1.06-1.13) |
| Cancer | 1.02 (0.99-1.05) | 1.02 (0.99-1.06) |
|  | Women | |
| All-cause | 1.06 (1.03-1.09) | 1.09 (1.06-1.12) |
| Cardiovascular disease | 1.10 (1.05-1.15) | 1.14 (1.09-1.20) |
| Cancer | 1.03 (0.99-1.07) | 1.04 (1.00-1.09) |
|  | Men | |
| All-cause | 1.01 (0.99-1.04) | 1.04 (1.01-1.06) |
| Cardiovascular disease | 1.03 (0.99-1.07) | 1.07 (1.02-1.11) |
| Cancer | 1.00 (0.96-1.05) | 1.01 (0.96-1.05) |

^1^Adjusted for offspring and parental birth cohort, parental and offspring zheight and parental sex

Abbreviations: BMI= body mass index

**Supplementary table 8.** Associations without height adjustment between zBMI at age 7 and adult mortality: hazard ratios per zBMI estimated from conventional analyses of own zBMI and from analyses using offspring zBMI at age 7 as instrumental variable (IV)

|  | Models without height adjustment^1^ | | |
| --- | --- | --- | --- |
| Cause of death | Conventional (C) | zBMI at age 7 as IV (IV) | P_IV vs C_^2^ |
| All-cause | 1.03 (1.01-1.05) | 1.07 (1.00-1.14) | 0.225 |
| Cardiovascular disease | 1.05 (1.01-1.09) | 1.14 (1.02-1.28) | 0.123 |
| Cancer | 1.04 (1.00-1.07) | 1.03 (0.93-1.14) | 0.905 |

^1^Adjusted for offspring and parental birth cohort and parental sex

^2^P-values from Durbin-Wu-Hausman test

Abbreviations: BMI= body mass index, IV=instrumental variable

**Supplementary table 9.** Associations without height adjustment between zBMI at age 13 and adult mortality: hazard ratios per zBMI estimated from conventional analyses of own zBMI and from analyses using offspring zBMI at age 13 as instrumental variable (IV)

|  | Models without height adjustment^1^ | | |
| --- | --- | --- | --- |
| Cause of death | Conventional (C) | zBMI at age 13 as IV (IV) | P_IV vs C_^2^ |
| All-cause | 1.05 (1.03-1.07) | 1.15 (1.07-1.22) | 0.006 |
| Cardiovascular disease | 1.13 (1.09-1.17) | 1.27 (1.14-1.42) | 0.023 |
| Cancer | 1.03 (1.00-1.07) | 1.05 (0.95-1.16) | 0.742 |

^1^Adjusted for offspring and parental birth cohort and parental sex

^2^P-values from Durbin-Wu-Hausman test

Abbreviations: BMI= body mass index, IV=instrumental variable

**Supplementary table 10.** Associations between zBMI at age 7 and adult mortality: hazard ratios per zBMI estimated from conventional analyses of own zBMI and from analyses using offspring zBMI at age 7 as instrumental variables (IV)

|  | Models^1^ | | |
| --- | --- | --- | --- |
| Cause of death | Conventional (C) | zBMI at age 7 as IV (IV) | P_IV vs C_ |
| Coronary heart disease | 1.09 (1.05-1.14) | 1.29 (1.13-1.48) | 0.012 |
| Respiratory disease | 1.06 (1.02-1.11) | 1.33 (1.16-1.53) | <0.001 |
| Digestive disease | 0.99 (0.93-1.05) | 1.06 (0.88-1.29) | 0.451 |
| Urogenital disease | 1.02 (0.92-1.14) | 1.31 (0.94-1.83) | 0.122 |
| Infectious disease | 1.12 (1.03-1.23) | 1.29 (0.99-1.68) | 0.277 |
| Nervous system disease | 1.03 (0.93-1.15) | 0.99 (0.73-1.35) | 0.777 |
| Muscle disease | 1.11 (0.93-1.32) | 1.85 (1.01-3.39) | 0.082 |
| Endocrine disease | 1.15 (1.07-1.23) | 1.66 (1.35-2.05) | <0.001 |
| Mental disease | 1.01 (0.96-1.07) | 1.10 (0.93-1.30) | 0.322 |
| External causes | 1.08 (0.97-1.21) | 1.39 (0.94-2.06) | 0.195 |

^1^Adjusted for offspring and parental birth cohort, parental and offspring zheight and parental sex

^2^P-values from Durbin-Wu-Hausman test

Abbreviations: BMI= body mass index, IV=instrumental variable

**Supplementary table 11.** Associations between zBMI at age 13 and adult mortality: hazard ratios per zBMI estimated from conventional analyses of own zBMI and from analyses using offspring zBMI at age at age 13 as instrumental variable (IV)

|  | Models^1^ | | |
| --- | --- | --- | --- |
| Cause of death | Conventional (C) | zBMI at age 13 as IV (IV) | P_IV vs C_^2^ |
| Coronary heart disease | 1.22 (1.17-1.28) | 1.49 (1.30-1.70) | 0.018 |
| Respiratory disease | 1.11 (1.06-1.17) | 1.41 (1.23-1.61) | <0.001 |
| Digestive disease | 1.01 (0.95-1.08) | 1.14 (0.94-1.39) | 0.184 |
| Urogenital disease | 1.15 (1.03-1.30) | 1.66 (1.19-2.31) | 0.021 |
| Infectious disease | 1.18 (1.08-1.30) | 1.70 (1.31-2.21) | 0.003 |
| Nervous system disease | 1.10 (0.99-1.23) | 1.00 (0.74-1.36) | 0.502 |
| Muscle disease | 1.10 (0.90-1.33) | 1.44 (0.80-2.61) | 0.342 |
| Endocrine disease | 1.36 (1.26-1.46) | 1.86 (1.51-2.30) | 0.002 |
| Mental disease | 0.96 (0.91-1.02) | 1.11 (0.94-1.31) | 0.067 |
| External causes | 1.12 (0.99-1.28) | 1.35 (0.92-1.97) | 0.313 |

^1^Adjusted for offspring and parental birth cohort, parental and offspring zheight and parental sex

^2^P-values from Durbin-Wu-Hausman test

Abbreviations: BMI= body mass index, IV=instrumental variable

**Supplementary table 12** Associations between zBMI at age 7 and adult mortality by birth cohort: hazard ratios per zBMI estimated from conventional analyses of own zBMI and from analyses using offspring zBMI at age 7 as instrumental variables (IV).

|  | Models | | | | | | | | | |
| --- | --- | --- | --- | --- | --- | --- | --- | --- | --- | --- |
| Cause of death | Conventional of zBMI at age 7, stratified by parental birth cohort^1^ | | | | | zBMI at age 7 as IV, stratified by parental birth cohort^1,3^ | | | | |
|  | 1930-39 | 1940-49 | P-value^4^ | 1950-83 | P-value^4^ | 1930-39 | 1940-49 | P-value^4^ | 1950-83 | P-value^4^ |
| All-cause | 1.05 (1.02-1.08) | 1.02 (0.98-1.06) | 0.235 | 1.06 (0.99-1.13) | 0.845 | 1.11 (1.01-1.21) | 1.12 (1.00-1.26) | 0.997 | 1.30 (1.03-1.63) | 0.283 |
| CVD | 1.04 (0.99-1.09) | 1.10 (1.03-1.17) | 0.258 | 1.18 (1.04-1.34) | 0.073 | 1.16(1.01-1.34) | 1.26 (1.04-1.54) | 0.570 | 1.34 (0.86-2.09) | 0.498 |
| Cancer | 1.04 (1.00-1.09) | 1.04 (0.98-1.11) | 0.978 | 0.97 (0.87-1.09) | 0.295 | 1.04 (0.90-1.19) | 1.05 (0.88-1.26) | 0.910 | 1.20 (0.85-1.71) | 0.640 |
|  | Conventional of zBMI at age 7, stratified by offspring birth cohort^2^ | | | | | zBMI at age 7 as IV, stratified by offspring birth cohort^2,3^ | | | | |
|  | 1952-62 | 1963-73 | P-value^4^ | 1974-96 | P-value^4^ | 1952-62 | 1963-73 | P-value^4^ | 1974-96 | P-value^4^ |
| All-cause | 1.03 (1.00-1.07) | 1.05 (1.01-1.08) | 0.535 | 1.03 (0.97-1.10) | 0.794 | 1.13 (1.02-1.25) | 1.11 (1.00-1.23) | 0.757 | 1.18 (0.96-1.44) | 0.912 |
| CVD | 1.02 (0.97-1.07) | 1.10 (1.04-1.16) | 0.062 | 1.13 (1.02-1.26) | 0.101 | 1.14 (0.97-1.34) | 1.32 (1.11-1.56) | 0.144 | 1.02 (0.72-1.45) | 0.573 |
| Cancer | 1.03(0.98-1.09) | 0.98 (0.83-1.15) | 0.643 | 1.01 (0.91-1.12) | 0.605 | 1.08 (0.93-1.26) | 1.05 (1.00-1.10) | 0.333 | 1.30 (0.94-1.80) | 0.494 |
|  | Conventional of zBMI at age 13, stratified by parental birth cohort^1^ | | | | | zBMI at age 13 as IV, stratified by parental birth cohort^1,5^ | | | | |
|  | 1930-39 | 1940-49 | P-value^4^ | 1950-83 | P-value^4^ | 1930-39 | 1940-49 | P-value^4^ | 1950-83 | P-value^4^ |
| All-cause | 1.08 (1.05-1.11) | 1.05 (1.01-1.09) | 0.152 | 1.07 (1.00-1.16) | 0.672 | 1.22 (1.11-1.35) | 1.20 (1.07-1.35) | 0.586 | 1.25 (1.08-1.69) | 0.532 |
| CVD | 1.14 (1.09-1.20) | 1.17 (1.09-1.25) | 0.924 | 1.25 (1.09-1.43) | 0.325 | 1.36 (1.17-1.59) | 1.48 (1.21-1.80) | 0.490 | 1.17(0.77-1.80) | 0.567 |
| Cancer | 1.04 (0.99-1.09) | 1.03 (0.97-1.09) | 0.884 | 1.00 (0.89-1.13) | 0.500 | 1.05 (0.90-1.21) | 1.09 (0.91-1.31) | 0.887 | 1.28 (0.92-1.77) | 0.282 |
|  | Conventional of zBMI at age 13, stratified by offspring birth cohort^2^ | | | | | zBMI at age 13 as IV, stratified by offspring birth cohort^2,5^ | | | | |
|  | 1952-62 | 1963-73 | P-value^4^ | 1974-96 | P-value^4^ | 1952-62 | 1963-73 | P-value^4^ | 1974-96 | P-value^4^ |
| All-cause | 1.06 (1.02-1.10) | 1.08 (1.04-1.11) | 0.852 | 1.06 (0.99-1.13) | 0.541 | 1.14 (1.02-1.27) | 1.31 (1.19-1.45) | 0.051 | 1.19 (0.99-1.43) | 0.958 |
| CVD | 1.12 (1.06-1.19) | 1.17 (1.11-1.25) | 0.571 | 1.23 (1.10-1.38) | 0.198 | 1.30 (1.10-1.55) | 1.55 (1.31-1.84) | 0.053 | 1.03 (0.74-1.43) | 0.235 |
| Cancer | 1.02 (0.97-1.08) | 1.04 (0.99-1.10) | 0.459 | 1.01 (0.91-1.11) | 0.546 | 0.97 (0.82-1.14) | 1.17 (1.00-1.37) | 0.105 | 1.17 (0.88-1.56) | 0.298 |

^1^Adjusted for parental and offspring zheight, offspring birth cohort and parental sex

^2^Adjusted for parental and offspring zheight, parental birth cohort and parental sex

^3^ Scaled to parental zBMI at age 7 via the denominator

^4^P-value from Z test of birth cohort category to the left of the p-value column compared to the first birth cohort column

^5^ Scaled to parental zBMI at age 13 via the denominator

Abbreviations: BMI= body mass index, IV=instrumental variable

**Supplementary table 13.** Associations between childhood zBMI and adult mortality during three age periods: hazard ratios per zBMI estimated from conventional analyses of own zBMI and from analyses using offspring zBMI as instrumental variable (IV)

|  | Models^1^ | | | | | | | |
| --- | --- | --- | --- | --- | --- | --- | --- | --- |
| Cause of death | Conventional with zBMI at age 7 as the exposure | | | | zBMI at age 7 as IV^2^ | | | |
|  | <60 years | 60-70 years | >70 years | P-value^3^ | <60 years | 60-70 years | >70 years | P-value^3^ |
| All-cause | 1.03 (0.99-1.06) | 1.05 (1.01-1.09) | 1.04 (1.00-1.08) | 0.486 | 1.03 (0.92-1.15) | 1.22 (1.08-1.37) | 1.17 (1.04-1.33) | 0.630 |
| CVD | 1.05 (0.99-1.12) | 1.09 (1.02-1.16) | 1.05 (0.99-1.12) | 0.806 | 1.16 (0.95-1.42) | 1.34 (1.10-1.62) | 1.14 (0.94-1.38) | 0.721 |
| Cancer | 1.02 (0.96-1.08) | 1.04 (0.99-1.10) | 1.05 (0.99-1.12) | 0.374 | 0.91 (0.76-1.08) | 1.17 (0.98-1.39) | 1.12 (0.92-1.37) | 0.331 |
|  | Conventional with zBMI at age 13 as the exposure | | | | zBMI at age 13 as IV^4^ | | | |
|  | <60 years | 60-70 years | >70 years | P-value^3^ | <60 years | 60-70 years | >70 years | P-value^3^ |
| All-cause | 1.04 (1.01-1.08) | 1.08 (1.04-1.13) | 1.08 (1.04-1.13) | 0.552 | 1.11 (1.00-1.24) | 1.29 (1.15-1.44) | 1.29 (1.15-1.46) | 0.918 |
| CVD | 1.17 (1.09-1.24) | 1.16 (1.09-1.24) | 1.15 (1.08-1.22) | 0.629 | 1.23 (1.02-1.50) | 1.50 (1.25-1.80) | 1.37 (1.13-1.64) | 0.930 |
| Cancer | 0.99 (0.94-1.05) | 1.05 (0.99-1.11) | 1.05 (0.99-1.12) | 0.370 | 0.97 (0.81-1.15) | 1.15 (0.97-1.36) | 1.14 (0.94-1.37) | 0.793 |

^1^Adjusted for parental and offspring zheight and parental sex

^2^Scaled to parental zBMI at age 7 via the denominator

^3^p-value from test of the proportional-hazards assumption on the basis of Schoenfeld residuals

^4^Scaled to parental zBMI at age 13 via the denominator

Abbreviations: BMI= body mass index, IV=instrumental variable

A) Conventional model of zBMI at age 7 B) IV model with zBMI at age 7 as the IV


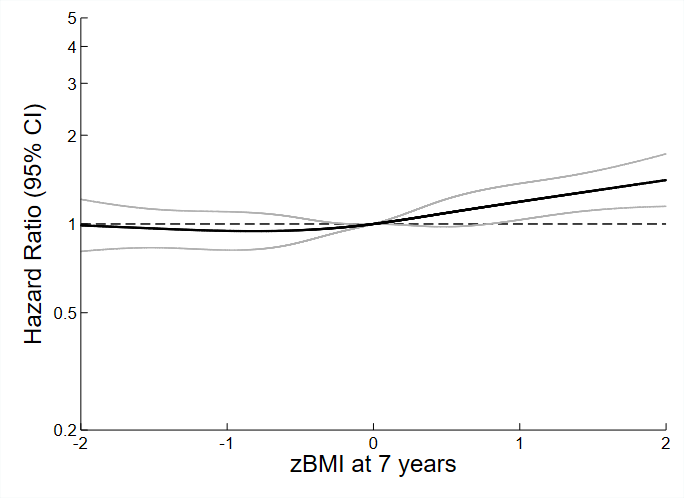

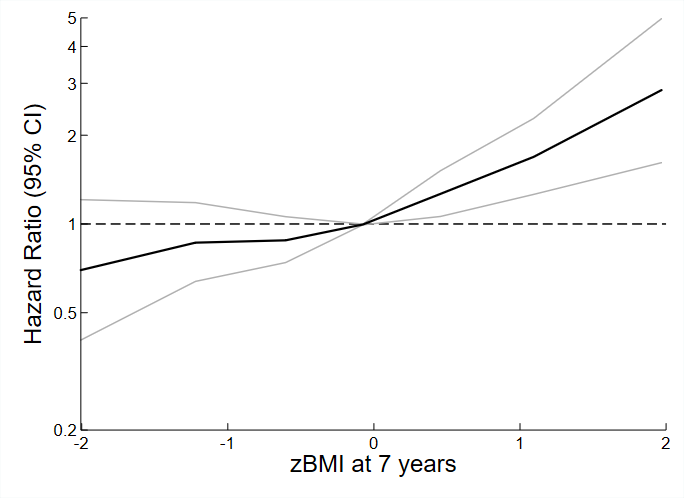


**Supplemental figure 8**. Association between zBMI at age 7 and cardiovascular mortality in women estimated by a conventional model and instrumental variable (IV) model. Non-linear conventional associations were modelled with restricted cubic splines with knots at the 5^th^, 27.5^th^, 50^th^, 72.5^th^ and 95^th^ percentiles. Non-linear IV associations were modelled by estimating local IV estimates in strata between approximately the 1.5, 10, 27.5, 50, 72.5, 90 and 98.5^th^ percentiles of the instrument-free exposure, which is the residual from when parental zBMI is regressed on offspring zBMI. Corresponding quantiles of the original exposure were used in piecewise linear plots made by joining the local IV estimates. All models are adjusted for maternal and offspring birth cohort, maternal and offspring zheight. 95% confidence intervals are indicated by the grey lines.

A) Conventional model of zBMI at age 7 B) IV model with zBMI at age 7 as the IV


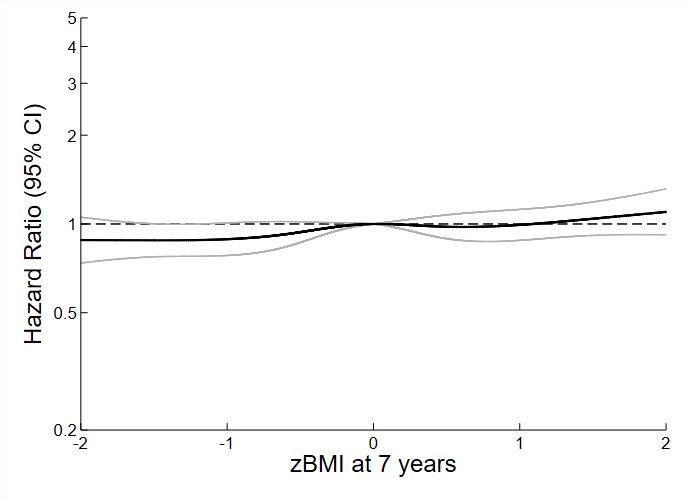

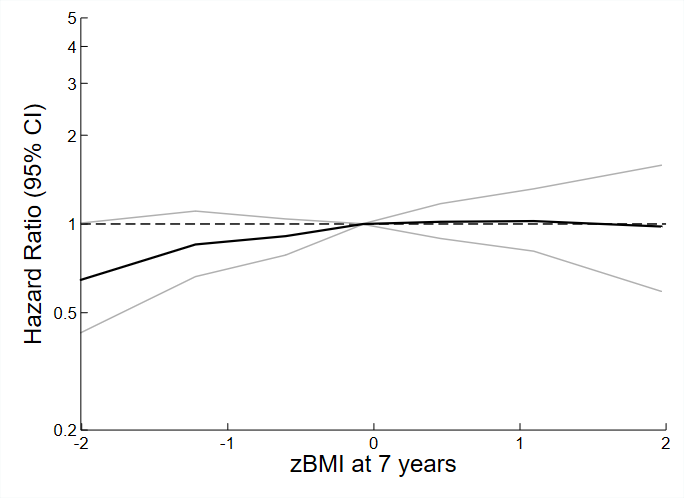


**Supplemental figure 9**. Association between zBMI at age 7 and cancer mortality in women estimated by a conventional model and instrumental variable (IV) model. Non-linear conventional associations were modelled with restricted cubic splines with knots at the 5^th^, 27.5^th^, 50^th^, 72.5^th^ and 95^th^ percentiles. Non-linear IV associations were modelled by estimating local IV estimates in strata between approximately the 1.5, 10, 27.5, 50, 72.5, 90 and 98.5^th^ percentiles of the instrument-free exposure, which is the residual from when parental zBMI is regressed on offspring zBMI. Corresponding quantiles of the original exposure were used in piecewise linear plots made by joining the local IV estimates. All models are adjusted for maternal and offspring birth cohort, maternal and offspring zheight. 95% confidence intervals are indicated by the grey lines.

A) Conventional model of zBMI at age 7 B) IV model with zBMI at age 7 as the IV


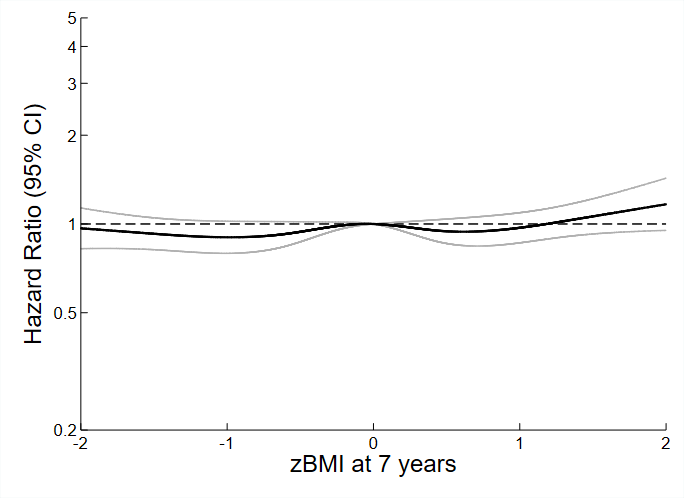

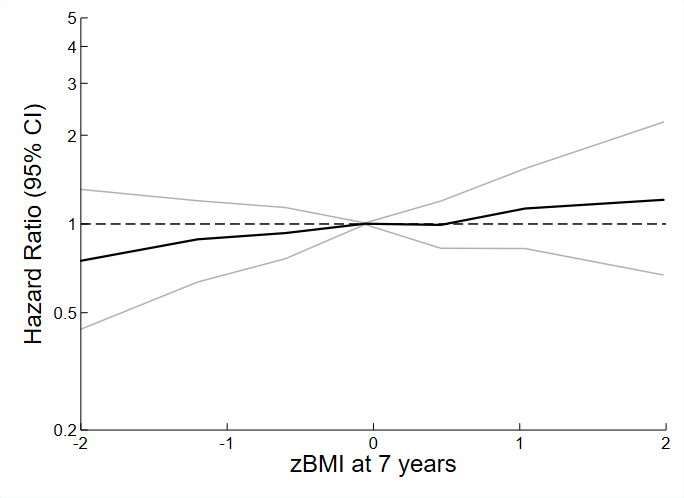


**Supplemental figure 10**. Association between zBMI at age 7 and cardiovascular mortality in men estimated by a conventional model and instrumental variable (IV) model. Non-linear conventional associations were modelled with restricted cubic splines with knots at the 5^th^, 27.5^th^, 50^th^, 72.5^th^ and 95^th^ percentiles. Non-linear IV associations were modelled by estimating local IV estimates in strata between approximately the 1.5, 10, 27.5, 50, 72.5, 90 and 98.5^th^ percentiles of the instrument-free exposure, which is the residual from when parental zBMI is regressed on offspring zBMI. Corresponding quantiles of the original exposure were used in piecewise linear plots made by joining the local IV estimates. All models are adjusted for maternal and offspring birth cohort, maternal and offspring zheight. 95% confidence intervals are indicated by the grey lines.

A) Conventional model of zBMI at age 7 B) IV model with zBMI at age 7 as the IV


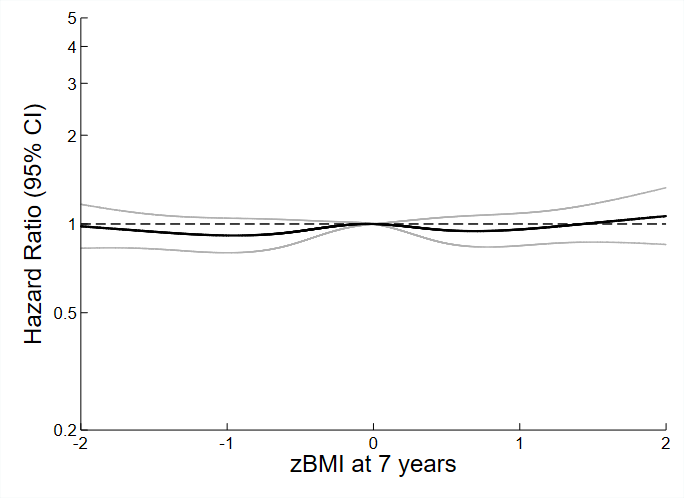

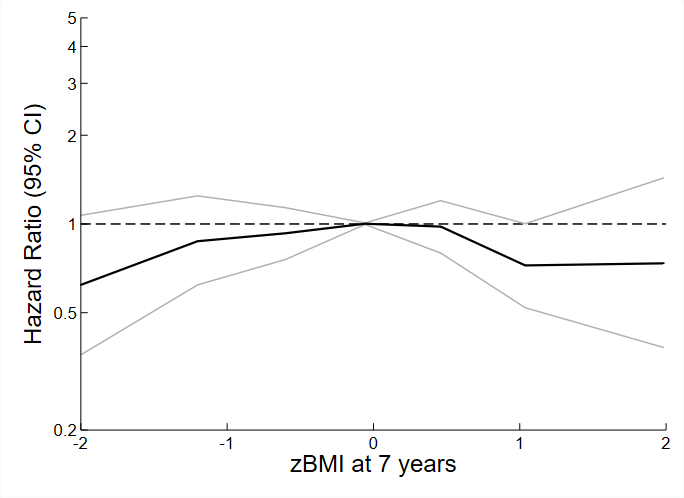


**Supplemental figure 11**. Association between zBMI at age 7 and cancer mortality in men estimated by a conventional model and instrumental variable (IV) model. Non-linear conventional associations were modelled with restricted cubic splines with knots at the 5^th^, 27.5^th^, 50^th^, 72.5^th^ and 95^th^ percentiles. Non-linear IV associations were modelled by estimating local IV estimates in strata between approximately the 1.5, 10, 27.5, 50, 72.5, 90 and 98.5^th^ percentiles of the instrument-free exposure, which is the residual from when parental zBMI is regressed on offspring zBMI. Corresponding quantiles of the original exposure were used in piecewise linear plots made by joining the local IV estimates. All models are adjusted for paternal and offspring birth cohort, paternal and offspring zheight. 95% confidence intervals are indicated by the grey lines.

A) Conventional model of zBMI at age 13 B) IV model with zBMI at age 13 as the IV


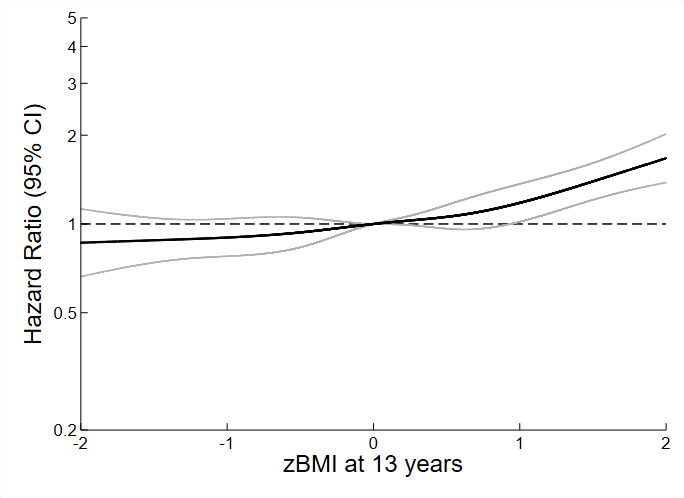

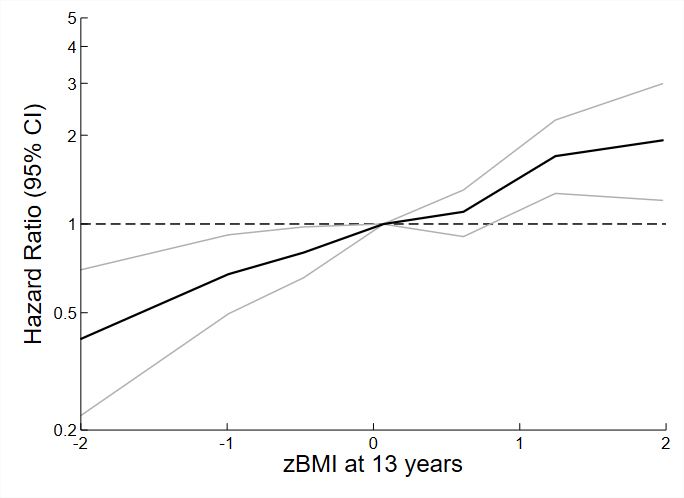


**Supplemental figure 12**. Association between zBMI at age 13 and cardiovascular mortality in women estimated by a conventional model and instrumental variable (IV) model. Non-linear conventional associations were modelled with restricted cubic splines with knots at the 5^th^, 27.5^th^, 50^th^, 72.5^th^ and 95^th^ percentiles. Non-linear IV associations were modelled by estimating local IV estimates in strata between approximately the 1.5, 10, 27.5, 50, 72.5, 90 and 98.5^th^ percentiles of the instrument-free exposure, which is the residual from when parental zBMI is regressed on offspring zBMI. Corresponding quantiles of the original exposure were used in piecewise linear plots made by joining the local IV estimates. All models are adjusted for maternal and offspring birth cohort, maternal and offspring zheight. 95% confidence intervals are indicated by the grey lines.

A) Conventional model of zBMI at age 13 B) IV model with zBMI at age 13 as the IV


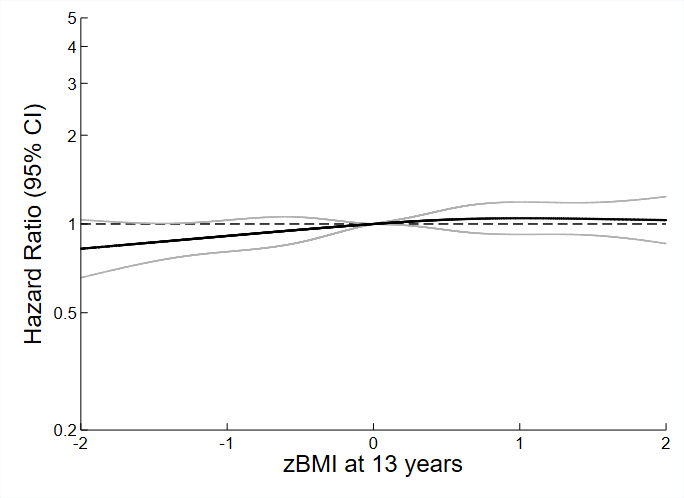

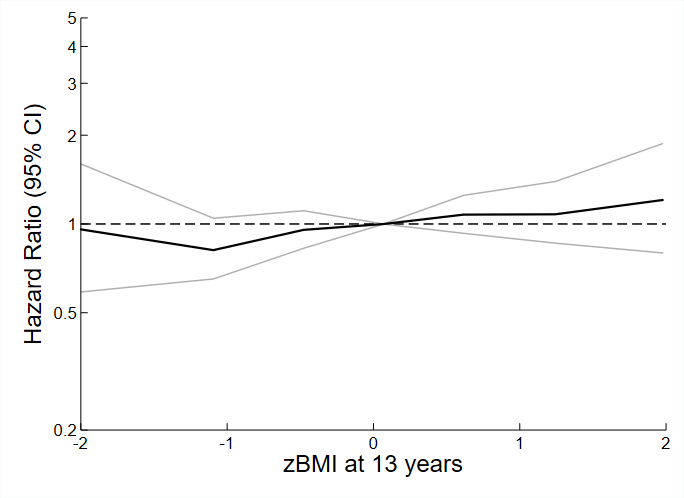


**Supplemental figure 13**. Association between zBMI at age 13 and cancer mortality in women estimated by a conventional model and instrumental variable (IV) model. Non-linear conventional associations were modelled with restricted cubic splines with knots at the 5^th^, 27.5^th^, 50^th^, 72.5^th^ and 95^th^ percentiles. Non-linear IV associations were modelled by estimating local IV estimates in strata between approximately the 1.5, 10, 27.5, 50, 72.5, 90 and 98.5^th^ percentiles of the instrument-free exposure, which is the residual from when parental zBMI is regressed on offspring zBMI. Corresponding quantiles of the original exposure were used in piecewise linear plots made by joining the local IV estimates. All models are adjusted for maternal and offspring birth cohort, maternal and offspring zheight. 95% confidence intervals are indicated by the grey lines.

A) Conventional model of zBMI at age 13 B) IV model with zBMI at age 13 as the IV


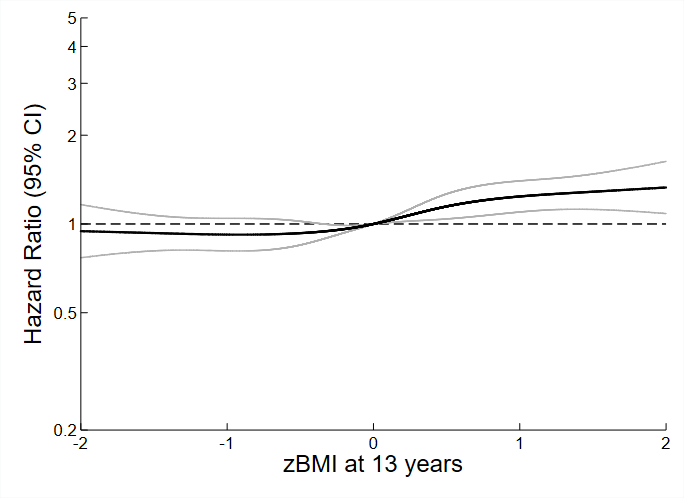

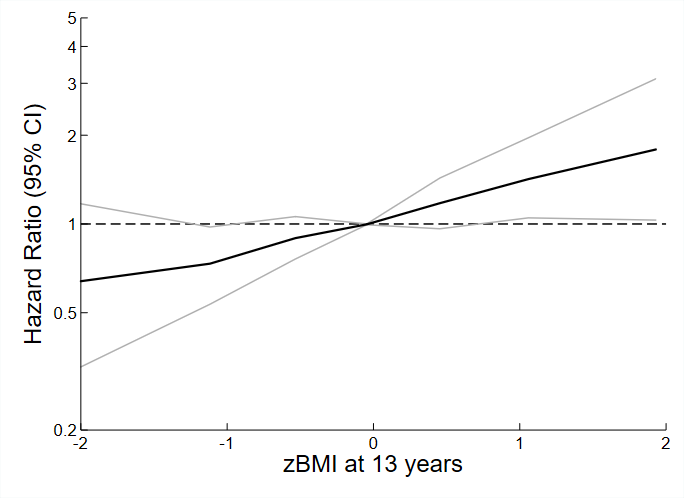


**Supplemental figure 14**. Association between zBMI at age 13 and cardiovascular mortality in men estimated by a conventional model and instrumental variable (IV) model. Non-linear conventional associations were modelled with restricted cubic splines with knots at the 5^th^, 27.5^th^, 50^th^, 72.5^th^ and 95^th^ percentiles. Non-linear IV associations were modelled by estimating local IV estimates in strata between approximately the 1.5, 10, 27.5, 50, 72.5, 90 and 98.5^th^ percentiles of the instrument-free exposure, which is the residual from when parental zBMI is regressed on offspring zBMI. Corresponding quantiles of the original exposure were used in piecewise linear plots made by joining the local IV estimates. All models are adjusted for maternal and offspring birth cohort, maternal and offspring zheight. 95% confidence intervals are indicated by the grey lines.

A) Conventional model of zBMI at age 13 B) IV model with zBMI at age 13 as the IV


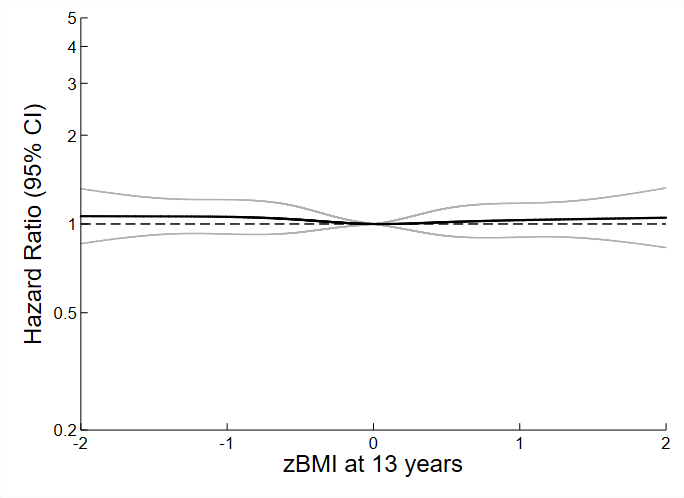

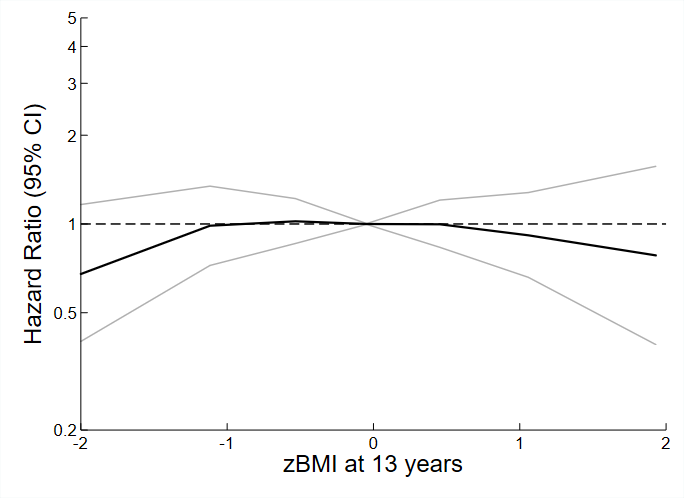


**Supplemental figure 15**. Association between zBMI at age 13 and cancer mortality in men estimated by a conventional model and instrumental variable (IV) model. Non-linear conventional associations were modelled with restricted cubic splines with knots at the 5^th^, 27.5^th^, 50^th^, 72.5^th^ and 95^th^ percentiles. Non-linear IV associations were modelled by estimating local IV estimates in strata between approximately the 1.5, 10, 27.5, 50, 72.5, 90 and 98.5^th^ percentiles of the instrument-free exposure, which is the residual from when parental zBMI is regressed on offspring zBMI. Corresponding quantiles of the original exposure were used in piecewise linear plots made by joining the local IV estimates. All models are adjusted for paternal and offspring birth cohort, paternal and offspring zheight. 95% confidence intervals are indicated by the grey lines.

**Supplementary table 14.** Associations between zBMI and all-cause mortality*:* hazard ratios (95 % confidence intervals) per zBMI below and above zBMI=0, respectively, estimated from conventional analyses of own zBMI and from analyses using offspring zBMI as an instrumental variable (IV)

|  | HR per zBMI below zBMI=0^1^ | | HR per zBMI above zBMI=0^1^ | |
| --- | --- | --- | --- | --- |
| Sex | Conventional model^2^ | IV model^2,3^ | Conventional model^2^ | IV model^2,3^ |
|  | zBMI at age 7 | | | |
| Men | 1.00 (0.95-1.05) | 1.13 (0.97-1.31) | 1.04 (0.98-1.10) | 0.96 (0.82-1.13) |
| Women | 1.02 (0.97-1.08) | 1.22 (1.08-1.38) | 1.11 (1.05-1.17) | 1.20 (1.06-1.37) |
|  | zBMI at age 13 | | | |
| Men | 0.98 (0.92-1.03) | 1.17 (1.01-1.37) | 1.08 (1.02-1.15) | 1.13 (0.96-1.33) |
| Women | 1.05 (0.98-1.11) | 1.33 (1.16-1.51) | 1.17 (1.10-1.23) | 1.27 (1.12-1.43) |

^1^In IV analyses, zBMI below or above zero is defined in relation to the residual from a linear regression where parental zBMI is regressed on offspring zBMI (approximately the 50^th^ percentile was used to define the two strata)

Adjusted for offspring and parental birth cohort, parental and offspring zheight

^2^Using offspring zBMI as the IV. The same age was used for the IV as for the exposure.

Abbreviations: BMI= body mass index, IV=instrumental variable

**Supplementary table 15.** Associations without height adjustment between zBMI at age 7 and adult mortality: hazard ratios per zBMI estimated from conventional analyses of own zBMI and from analyses using offspring zBMI at age 13 as instrumental variable (IV)

|  | Models^1^ | | |
| --- | --- | --- | --- |
| Cause of death | Conventional (C) | zBMI at age 13 as IV (IV)^2^ | P_IV vs C_^3^ |
| All-cause | 1.04 (1.02-1.06) | 1.25 (1.16-1.36) | <0.001 |
| Cardiovascular disease | 1.06 (1.03-1.10) | 1.42 (1.24-1.62) | <0.001 |
| Cancer | 1.04 (1.00-1.07) | 1.09 (0.96-1.23) | 0.401 |

^1^Adjusted for offspring and parental birth cohort and parental sex

^2^Scaled to parental zBMI at age 7 via the denominator

^3^P-values from Durbin-Wu-Hausman test

Abbreviations: BMI= body mass index, IV=instrumental variable

**Supplementary table 16.** Associations without height adjustment between zBMI at age 13 and adult mortality: hazard ratios per zBMI estimated from conventional analyses of own zBMI and from analyses using offspring zBMI at age 7 as instrumental variable (IV)

|  | Models^1^ | | |
| --- | --- | --- | --- |
| Cause of death | Conventional (C) | zBMI at age 7 as IV (IV)^2^ | P_IV vs C3_ |
| All-cause | 1.07 (1.04-1.09) | 1.14 (1.06-1.22) | 0.063 |
| Cardiovascular disease | 1.16 (1.11-1.20) | 1.23 (1.09-1.38) | 0.309 |
| Cancer | 1.03 (0.99-1.07) | 1.06 (0.95-1.19) | 0.578 |

^1^Adjusted for offspring and parental birth cohort and parental sex

^2^Scaled to parental zBMI at age 13 via the denominator

^3^P-values from Durbin-Wu-Hausman test

Abbreviations: BMI= body mass index, IV=instrumental variable

|  | Models^1^ | | |  |
| --- | --- | --- | --- | --- |
| Cause of death | Conventional | BMI in boys as IV | BMI in girls as IV | P_offspring sex interaction in IV model_^2^ |
| All-cause | 1.05 (1.02-1.08) | 1.07 (0.97-1.17) | 1.22 (1.10-1.35) | 0.109 |
| Cardiovascular disease | 1.07 (1.02-1.13) | 1.11 (0.95-1.29) | 1.35 (1.14-1.61) | 0.120 |
| Cancer | 1.05 (1.00-1.11) | 0.95 (0.82-1.10) | 1.20 (1.02-1.40) | 0.035 |

**Supplementary table 17.** Associations between zBMI at age 7 and adult mortality: hazard ratios per zBMI estimated from conventional analyses of own zBMI and from analyses using offspring zBMI in boys and girls at age 7 as separate instrumental variables (IV)

^1^Adjusted for offspring and parental birth cohort, parental and offspring zheight and parental sex

^2^P-values from Z-test of interaction term in the numerator used to obtain the IV estimate. The numerator was adjusted for offspring and parental birth cohort, parental and offspring zheight, parental and offspring sex

Abbreviations: BMI= body mass index, IV=instrumental variable

|  | Models^1^ | | |  |
| --- | --- | --- | --- | --- |
| Cause of death | Conventional | BMI in boys as IV | BMI in girls as IV | P_offspring sex interaction in IV model_^2^ |
| All-cause | 1.07 (1.03-1.10) | 1.20 (1.09-1.33) | 1.25 (1.13-1.37) | 0.709 |
| Cardiovascular disease | 1.16 (1.10-1.23) | 1.33 (1.13-1.55) | 1.41 (1.20-1.66) | 0.707 |
| Cancer | 1.04 (0.99-1.10) | 1.01 (0.87-1.17) | 1.17 (1.00-1.35) | 0.149 |

**Supplementary table 18.** Associations between zBMI at age 13 and adult mortality: hazard ratios per zBMI estimated from conventional analyses of own zBMI and from analyses using offspring zBMI in boys and girls at age 13 as separate instrumental variables (IV)

^1^Adjusted for offspring and parental birth cohort, parental and offspring zheight and parental sex

^2^P-values from Z-test of interaction term in the numerator used to obtain the IV estimate

Abbreviations: BMI= body mass index, IV=instrumental variable
